# Scanpath visualization and comparison using visual aggregation techniques

**DOI:** 10.16910/jemr.10.5.9

**Published:** 2018-01-08

**Authors:** Vsevolod Peysakhovich, Christophe Hurter

**Affiliations:** ISAE-SUPAERO , France; ENAC, ToulouseFrance

**Keywords:** eye tracking, scanpath, saccades, visualization, fixation clustering, mean-shift, edge bundling, flow directional map, oriented line integral convolution

## Abstract

We demonstrate the use of different visual aggregation techniques to obtain non-cluttered visual representations of scanpaths. First, fixation points are clustered using the mean-shift algorithm. Second, saccades are aggregated using the Attribute-Driven Edge Bundling (ADEB) algorithm that handles a saccades direction, onset timestamp, magnitude or their combination for the edge compatibility criterion. Flow direction maps, computed during bundling, can be visualized separately (vertical or horizontal components) or as a single image using the Oriented Line Integral Convolution (OLIC) algorithm. Furthermore, cosine similarity between two flow direction maps provides a similarity map to compare two scanpaths. Last, we provide examples of basic patterns, visual search task, and art perception. Used together, these techniques provide valuable insights about scanpath exploration and informative illustrations of the eye movement data.

## Introduction

The affordable prices of modern eye tracking devices and the maturity of analytical methods have made gaze recordings a standard source of information when studying human-computer interaction, user behavior or cognition [ 14, 22 ]. Gaze positions are computed at high speed (up to 2 kHz) with additional data dimension like pupil diameter; and are further processed to analyze the behavior of users. This analysis can be supported by a statistical comparison of numerous metrics derived from eye movements (e.g. fixation duration, saccade amplitude etc.) or static, dynamic and interactive visualizations. Gaze record processing in the data space [ 18 ] is more popular than processing in the image space and displaying the data using visual simplification techniques. However, interest has recently grown in image-based techniques due to their fast computation and their efficiency to support a visual analysis [ 19 ].

Raw eye tracking data is complex, and, therefore, needs to be simplified for a visual analysis to support an efficient exploration of visual patterns. A heat or saliency map [ 38 ] – a conventional visualization of fixation distribution – allows an analyst to instantly perceive what elements of the scene were focused on. Gaze plots – classic scanpath visualizations – represent fixation points as circles with the diameter proportional to fixations duration and connected with straight lines. However, in general, such visualizations rapidly become cluttered after a dozen drawn saccades. Therefore, scanpath analysis and comparison, a cumbersome task, is often solved at a higher level [ 28 ] implying analyst-defined areas of interests (AOIs) and visual analysis using infographics such as line and bar charts, scatter plots, timeline visualizations, histograms etc. [ 2 ]. Nevertheless, to the best of our knowledge, there does not yet exist a commonly accepted visualization technique for scanpaths in an intermediate state between raw data and high-level representation.

Among techniques for visual simplifications of graphs, edge bundling [ 25 ] has exhibited a high potential to support gaze analysis [ 33, 40, 26, 21 ]. Considering a recorded gaze path as a sequence of points (i.e. fixations) connected by lines (i.e. saccades), the resulting visualization of these data corresponds to a set of tangled lines. Edge bundling techniques aggregate these lines into bundles using a compatibility criterion which is often defined as the line vicinity: close lines are aggregated to create an aggregated path.

A recent review of state-of-the-art eye-tracking data visualizations [ 2 ] revealed that, in spite of an important number of high-quality visualization techniques available to eye tracking practitioners, there is still a lack of efficient point-based scanpath visualizations. For example, Hurter et al. [ 21 ] proposed applying edge bundling to eye traces. Peysakhovich et al. [ 33 ] noted the importance of the saccade direction and developed an edge bundling framework that allows to take account of the orientation of edges. Based on these ideas, in this paper, we present a new rationale for scanpath visualizations using visual aggregation techniques that make it possible to reduce visual clutter and provide a mathematical base for scanpath comparison. The paper is structured as follows: after a brief review of previous work on eye-tracking visualizations, we explain our design rationale consisting of four steps: fixation detection, fixation clustering, saccade bundling, and generation of flow direction maps; then we explain a set of examples where the visual aggregation techniques help to extract meaningful information. Finally, we present an example for comparing the scanpaths of two participants using a similarity map. This work contributes to the state-of-the-art eye tracking visualizations techniques describing in detail how to reduce clutter in visual scanpath visualizations.

## Previous work

Fixation patterns can be transformed into transitions between meaningful semantically different AOIs that can be analyzed using graphs, trees, or matrices [ 4 ]. The sequences of annotated fixations can be further compared using string edit metrics [ 27, 28, 15 ], or represented as a dotplot to discover scanpath patterns using linear regression and hierarchical clustering [ 16 ]. The string-based scanpath comparison can also be performed without an a priori AOI definition by regrouping fixations into clusters automatically [ 13, 37 ].

Various visualizations exist to support the exploration of the gaze data such as color bands [ 8 ], eye movements plots [ 6 ], radial AOI transition graphs [ 3 ], saccade plots [ 7 ], AOI rivers [ 9 ], or interactive systems [ 35, 31 ].

Scanpaths can also be broken down into individual saccades that can be compactly represented as radial plots [ 17 ], or compared numerically using vector-based alignment and statistical comparison of an average saccade [ 23 ].

## Methodology

In this section, we describe the pipeline for the generation of a scanpath visualization using visual aggregation techniques. First, fixations and saccades are extracted from the gaze recording. Then, fixations are clustered and saccades are bundled together. Finally, the analysis of gaze data is performed using a flow visualization map.

### Fixation detection

**Figure 1:**
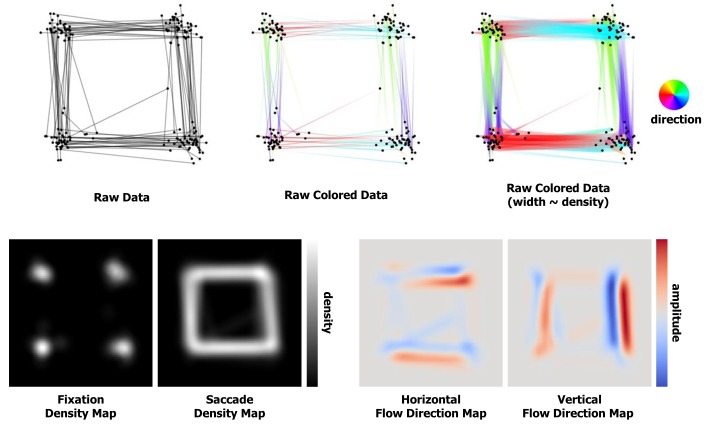
Different representations and maps of the raw data.

A typical gaze recording consists of horizontal and vertical coordinates varying over time. In order to apply an edge bundling technique, we have to define the control points – the start and end points of trails that are not affected by the edge aggregation. The trivial choice for the gaze data are fixations. Fixations can be detected from the raw data using dispersion or velocity thresholds [ 1, 32, 36 ]. The consecutive fixations are connected with straight lines that represent saccades. Hence, in terms of graph theory, eye movement data can be represented as a directed graph where fixations are vertices and saccades are edges (see Figure 1, raw data). Note that throughout this paper we call this representation (fixations connected with saccades) “raw data” – raw meaning relative to the application of the visual aggregation techniques – the focus of this work.

### Fixation clustering

**Figure 2:**
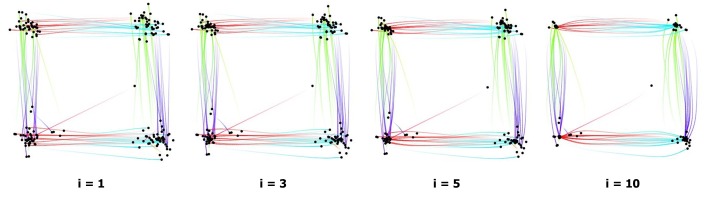
Clustering of fixations using the mean-shift algorithm (i = #iterations).

When we fixate the exact same object multiple times, the detected fixation points are rarely at the exact same position due to the inaccuracy of video-based eye tracking systems and the size of the fovea. Therefore, while semantically equal, the spread of the fixation points produces unnecessary visual clutter. Fixation clustering algorithms can reduce the clutter by aggregating adjoining fixations. In this work, we propose applying the mean-shift algorithm [ 11 ]. This uses kernel density estimation to generate a density map; the points are then iteratively shifted to their densest neighborhood. The density map of fixations is equal to a saliency map (Figure 1, bottom left), i.e. for N fixations at positions {x_n,n=(1,N)‾ } the density map is defined by Equation 1 where K(∙) is a bivariate radial kernel and δ is the Kronecker symbol. In this work, we implemented maps with a resolution of 420 × 420 and a kernel width of 31. One map pixel corresponds to a 4 × 4 pixel square on the screen. In each iteration, points are shifted towards the locally densest area, and the density map is then recomputed. To compute this gradient, we use a neighborhood width of 40. We performed 10 clustering iterations for all paper illustrations. Figure 2 illustrates a few intermediate results of the fixation clustering. The parameters (number of iterations, kernel size, map resolution etc.) have been chosen empirically. Some parameters are related, for example, the kernel size and the gradient size (gradient should be higher than the kernel size), and some parameters must be adapted according to the recorded data (for instance, the map resolution can be decreased if the viewed objects are placed far enough from each other). For consistency and comparison purposes, we fixed the same parameters for every generated image.

**Figure:**
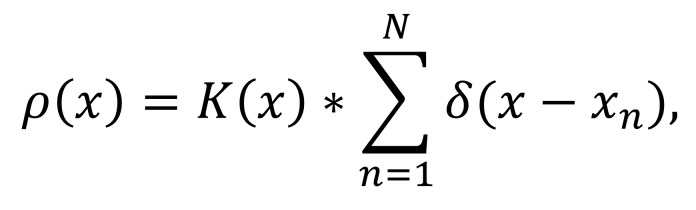


### Saccade bundling

Diminishing the dispersion of fixation points around a focused location reduces visual clutter. It also facilitates the use of the edge bundling technique by moving the control points closer to each other (which are not affected by the edge aggregation). Edge bundling techniques regroup the close edges and draw them in bundles. Visual suppression during saccades (i.e. the absence of information encoding [ 29 ]) supports such an approach. The lines that represent the saccades do not carry any information apart from connecting the subsequent fixations. Many edge bundling algorithms exist; few, however, handle the orientation of the edge (saccades). In this we use the Attribute-Driven Edge Bundling (ADEB) framework [ 33 ]. This is an extension of the Kernel Density Estimation Edge Bundling (KDEEB) method [ 20 ], which applies the mean-shift algorithm to resampled edges. In comparison to previous work [ 33 ], we provide additional uses of the ADEB framework and open eye tracking datasets for which this tech-nique helps to understand recorded data. Furthermore, we have taken Peysakhovich et al. work further by using the underlying computed gradient map (flow direction map) presented in the next section

The method is similar to the procedure described in the Section “Fixation clustering”, except for resampling the lines (saccades) that connect fixation points and computing the density map taking into account all these resampled points (see Figure 1, bottom, Fixation density map vs. Saccade density map). ADEB also introduced the flow direction maps – vector fields generated similar to density maps by weighting a unit vector tangent to the saccade curve with a bivariate radial kernel. Given the N fixations, the resampling of the N− 1 saccades gives the points {s_m,m=(1,M_n )‾ }, where M_n is the number of points composing the n-th saccade. Thus, the flow direction map is defined by Equation 2 s_(m+1)-s_m being an estimate of the tangent vector to the saccade curve at the sampling point s_m. In the presence of a dominant local direction, the directional component is significant, otherwise, the vector sum of the directions is relatively small (Figure 1, bottom right). At each point, a local subspace of compatible directions is defined as the cosine similarity between the edge direction and the flow direction at this point, i.e. it is defined by a maximum allowed angle between two vectors. The gradient of advection is not computed across all points in the neighborhood as in standard mean-shift, but across the subneighborhood that is compatible directionally. We used the same parameters for the map size, kernel width and neighborhood width as for fixation clustering, and 60° for the compatibility criteria.

**Figure:**
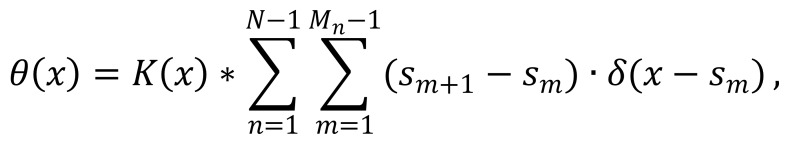


ADEB introduced a compatibility criterion which is based on the edges proximity and direction: close edges of the same direction are aggregated. However, other factors can be considered, for example, the temporal dimension, or the length of the saccade. We illustrate the use of these different factors in the art perception example.

We performed 20 saccade bundling iterations for all paper illustrations. Figure  shows a few iterations of the saccades bundling. Similar to the number of fixation clustering iterations, the number of bundling iterations for the saccades was chosen arbitrarily but seemed appropriate for the goal of this work. Performing more iterations would simply refine the flow direction maps further and shift the compatible saccades closer together.

**Figure 3:**
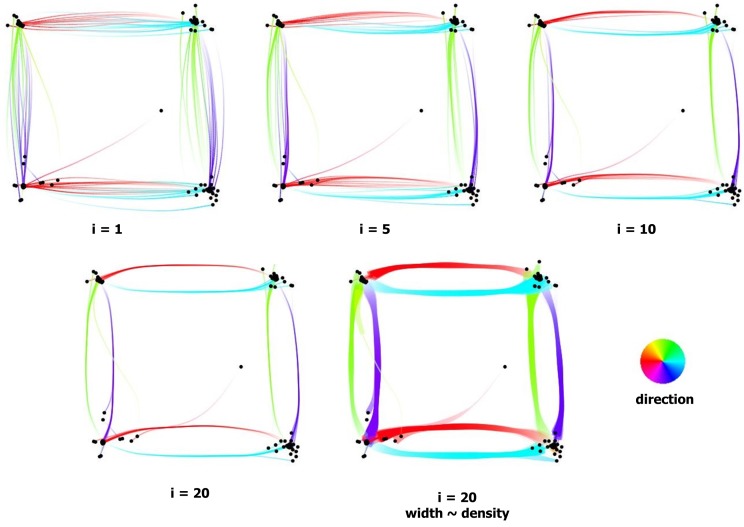
Bundling of saccades using the Attribute-Drive Edge Bundling algorithm (i = #iterations). Line width can be set proportional to the edge density.

### Flow direction map visualization

**Figure 4:**
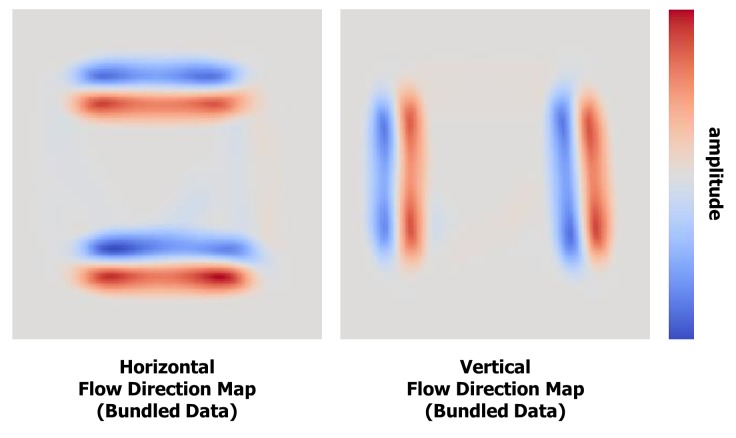
Visualization of the horizontal (on the left) and vertical (on the right) components of the flow direction map of the square scanpath after fixation clustering and saccades bundling

The flow direction map is implemented as two floatingpoint textures corresponding to horizontal and vertical components (Figure 4). In the ADEB framework [ 33 ] these textures are used only to define the edge compatibility criterion. However, the visual analysis of the flow direction map can round off the exploration of the bundled saccades traces to identify the clearly visible saccade patterns. Comparing, for instance, the maps before (Figure 1, bottom right) and after (Figure 4) applying the saccade bundling algorithm shows how the vertical and horizontal paired transitions become clearly visible. Nevertheless, while exploring two separate components can be intuitive when the flows are parallel to the components (i.e. purely vertical or horizontal, as in the square scanpath dataset), it is more troublesome in cases of diagonal or circular flows where both components are non-null. In the scientific visualization domain a variety of methods exist that can depict a vector field in a single 2D image [ 34 ]. Flow visualization techniques include direct flow visualizations using arrow glyphs, geometric flow visualizations using streamlines, feature-based flow visualizations using topological information, and dense, texturebased flow visualizations using repetition of a texture according to the local flow vector [ 24 ]. The texture-based flow visualization is among the most versatile and effective methods, and is easy to implement.

**Figure 5:**
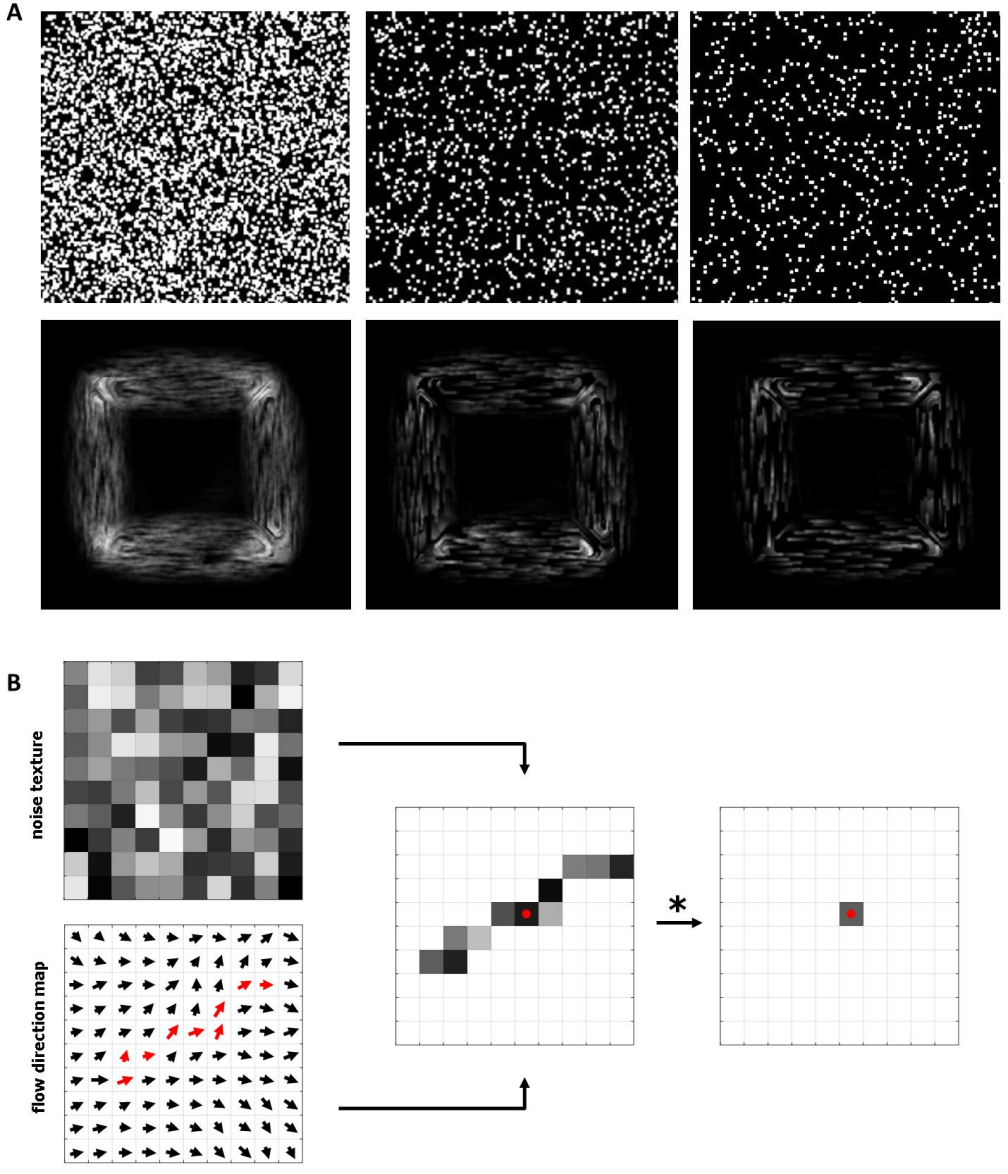
A) Visualization of the flow direction map for the square scanpath dataset using the oriented line integral convolution algorithm. In the top row, three input textures of decreasing density are shown. In the bottom row, the corresponding OLIC visualizations are depicted. B) For each pixel, a noise texture is filtered using a convolutional kernel according to the flow direction map.

In this work, we use the Line Integral Convolution (LIC) algorithm [ 10 ] which filters an input texture along streamlines using a one-dimensional convolutional kernel (for instance, a simple constant or Gaussian kernel). Using white noise textures as an input (Figure 5a, top row), LIC visualizes vector fields where ink droplets follow the flow direction. The intensity I(χ) of a pixel at location x is calculated by Equation 3 where k(∙) is the convolution kernel, T(∙) is the input noise texture, and s(∙) is the function that parametrizes the streamlines of the flow direction map. To each pixel at position y it associates one of the surrounding pixels according to the direction vector at that location (Figure 5b).

**Figure:**
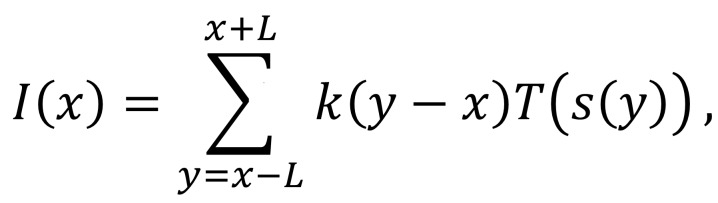


By using a sparse noise texture and ramp-like kernel function as an input, Oriented LIC (OLIC) [ 39 ] enables visual separation of streamlines with the same direction but opposite orientation in static images. The ramp-like kernel function makes the ink intensity vary according to the streamline, indicating the direction of the flow (Figure 5a, bottom row). By phase-shifting the kernel, these textures can be animated to indicate the flow direction more clearly.

### Illustrations Datasets

We considered three use cases: a square scanpath, a visual search task and an art perception task. A participant’s gaze position was recorded at 500 Hz with a remote SMI RED eye tracker (SensoMotoric Instruments GmbH, Germany). A 9-point calibration was performed in the beginning of the acquisition. The calibration was validated with four additional fixation points until the precision was below 1°. The participants had a viewing distance of approximately 60 cm from the 22-inch LCD monitor with 1680 × 1250 pixels screen resolution. The fixations and saccades were detected using the Event Detector 3.0.20 by SMI using default settings. The software that generated the illustrations using the described visual aggregations algorithms was implemented in C#. All the datasets, containing x and y coordinates of the start and end fixation points of each saccade and their timestamp, are available in supplementary files.

**Square scanpath.** In this example, the participant followed a small black circle on the screen for one minute. The circle moved from corner to corner of the square, each side of which has a length of 200 pixels. During the first half of the trial, the circle moved in the clockwise direction, during the other half it moved anticlockwise. The resulting dataset contains 90 saccades.

**Visual search task.** During this task, the participant had to find all the numbers from 1 to 90 in the correct order. This test was used in the Soviet Union to test children’s attentional capabilities. We considered the first minute of the task recording. The resulting dataset contains 595 saccades.

**Art perception.** The participant freely observed three paintings for one minute each. The participant was presented with Café Terrace at Night (1888) by Vincent van Gogh, I and the Village (1911) by Marc Chagall, and The Creation of Adam (1510) by Michelangelo. The resulting datasets contains 320, 380 and 375 saccades respectively.

## Results and Discussion

In this section, we present and discuss the three use cases to illustrate the described scanpath visualizations using visual aggregation techniques, i.e. fixation clustering and saccade bundling. We close the discussion with an example of scanpath comparison using the flow direction maps.

### Square scanpath

This basic square scanpath illustrates all the steps of the described visualization methods. Figure 1 shows the raw fixations and saccades. At the top, fixations are represented as small black dots and saccades are shown with different color encodings. The color coding of the saccade direction gives us initial information about the scanpath nature. We used a standard rainbow colormap. Though far from perfect, and confusing for the viewer in some situations [ 30, 5 ], we consider it suitable for the illustrations presented here. Indeed, for the purpose of illustration we needed at least four principal colors to depict four compass directions. For example, we can easily distinguish red-cyan horizontal and green-violet vertical transitions in Figure 3. Representing the line width proportionally to the local density facilitates the reading of the colors. Based on the raw fixations and saccades, four maps (2D textures) are generated: a fixation density map to perform fixation clustering, a saccade density map and a flow direction map to perform saccade bundling. We used a grayscale colormap for the density maps and the diverging colormap proposed by Moreland [ 30 ] for the flow direction maps.

The square scanpath illustrates the inherent visual clutter of gaze recordings. While the target presented a small dot appearing at the exact same locations, the fixations were detected at quite different positions. A few iterations of fixation clustering make it possible to bring adjacent fixations together (Figure 2), and saccade bundling merges the saccades of the same direction and orientation (Figure 3). After applying these two steps, we can easily distinguish mutual transitions between corners. By separating the edges of different directions, the flow direction map of bundled data can also be used to interpret the data (Figure 4). While the overlapping saccades of the raw data canceled the flow of the opposite orientation (Figure 1, bottom right), the bundled layout has eight clearly visible flows corresponding to saccade bundles: four horizontal (Figure 4, left) and four vertical (Figure 4, right).

The resulting flow direction map can be shown as a single texture by using the OLIC technique. Figure 5a shows the result of convolving noisy textures with the flow direction map from Figure 4. The ink droplets of varying intensity that follow the saccade flow allow instant reading of the flow direction and orientation.

### Visual search task

**Figure 6:**
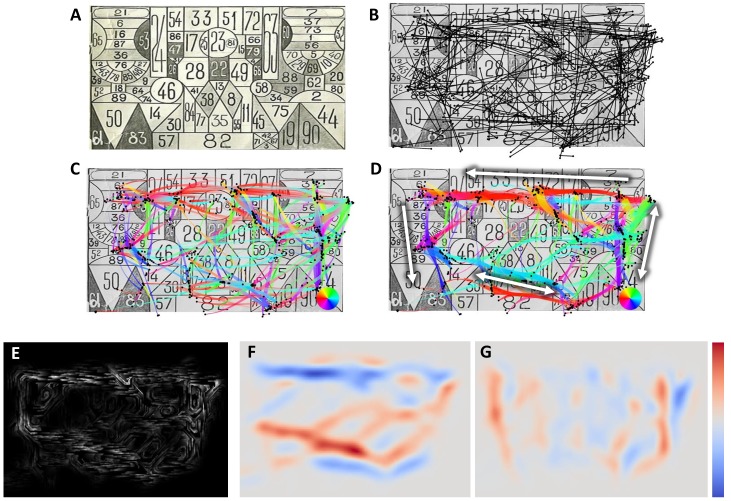
The scanpath visualization for the visual search task. A) visual stimulus, B) raw fixations and saccades, C) clustered data colored with line width proportional to local density, D) bundled data with line width proportional to local density, E) OLIC image of the flow direction map, F) horizontal and G) vertical components of the flow direction map.

In this example (Figure 6a and 6b), we can notice the benefit of the proposed scanpath visualization when hundreds of saccades are present. While the clustered layout with the color and line width encoding already gives us a few insights about the direction of the scanpath (Figure 6c), the clustered and bundled layout significantly reduces the visual clutter and uncovers the circular scanpath (Figure 6d). The red east-west transitions at the top, the blue-violet north-south transitions on the left, the cyan west-east transition at the bottom, and the green southnorth transitions on the right can be seen. We can also spot the clearly visible violet north-south transition on the right and red east-west transition at the bottom. These latter transitions indicate the presence of local loops that can be seen on the OLIC representation (Figure 6e). The participant confirmed the circular visual search strategy afterwards. The obtained insights can also be seen in the horizontal and vertical components of the flow direction map (Figure 6e and 6g).

### Art perception

**Figure 7:**
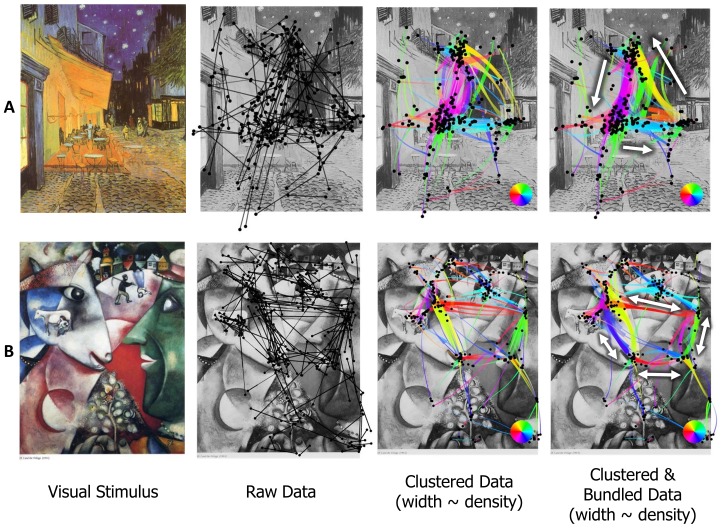
The scanpath visualization for the Vincent van Gogh (A) and Marc Chagall (B) paintings.

As in the visual search task example, the visualization of the art perception datasets reveals the scan strategy used by the participant viewing the masterpieces. Figure 7a shows that the participant explored the Vincent van Gogh painting in a triangle between the café terrace, the night sky and the shop on the street corner. The line width encoding according to the bundle density also tells us that the least seen element was the corner shop, and the majority of transitions were between the blue sky and the yellow terrace. Figure 7b uncovers the main transitions between the eyes and the lips of the peasant and the cow. Small transitions to the figures of two peasants on the top of the painting are also easily visible in the proposed scanpath representation.

**Figure 8:**
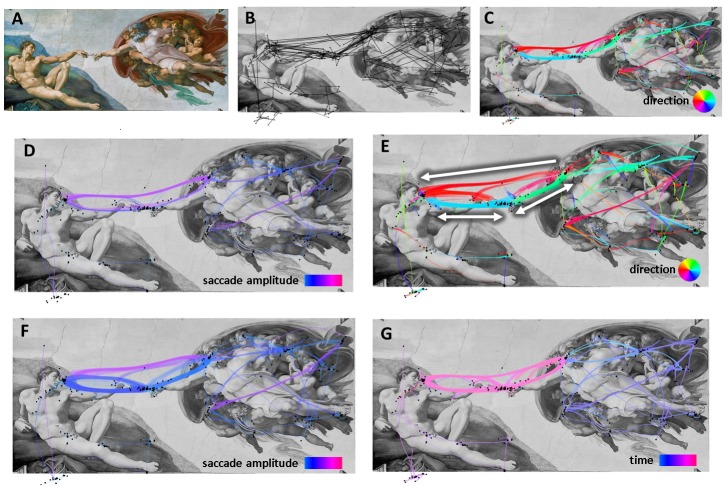
scanpath visualization of the dataset for the Michelangelo painting. A) visual stimulus, B) raw fixations and saccades, C) data bundled according to saccade direction, D) layout “C” colored according to saccade length, E) data bundled according to both direction and saccade length, F) layout “E” colored according to saccade length, G) layout “C” colored according to timestamp.

Figure 8 shows the bundled layout and different color encodings of the gaze of the participant exploring the Michelangelo masterpiece. Figure 8c shows that the bundled layout reveals the main transitions between Adam’s head and hand and God’s head and hand. However, Figure 8d shows the color encoding according to the saccade amplitude, and reveals that saccades having the same direction between the heads and hands were bundled together with the transitions between faces. We can easily correct this by applying multi-criteria bundling using both direction and saccade amplitude as a compatibility criterion. The resulting layout (Figure 8e) separates Adam’s hand-face and God’s face-hand transitions from the God-Adam face transitions. Encoding saccade magnitude (Figure 8f) allows us to see that the bundles take the differences of the saccade length into account. Moreover, color coding the saccade timestamp (Figure 8g) shows us the order in which the elements were looked at: first, the figures around God, next, Adam’s body, and, at last, a long exploration of the main characters’ faces and hands transitions.

### Scanpaths comparison

**Figure 9:**
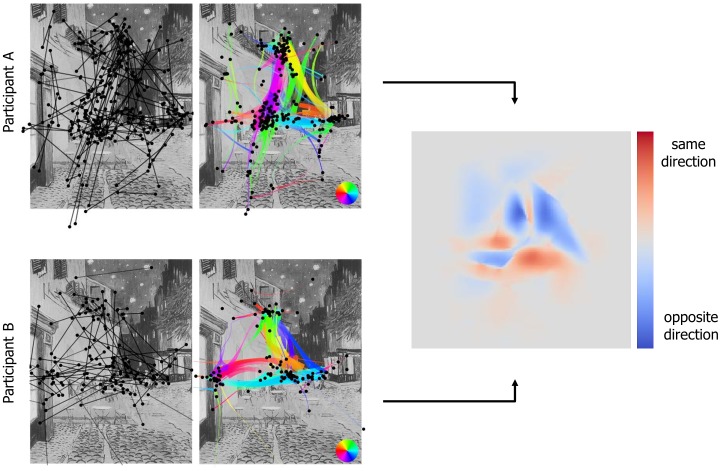
Comparison of scanpaths of two participants who observed the Vincent van Gogh painting. The similarity map is given by cosines between the two flow direction maps.

The techniques presented provide a visual support for an analysis. Nevertheless, the rationale also provides us with flow direction maps which allow us to not only visualize but also quantitatively compare. Le Meur and Baccino [ 28 ] presented a number of methods for comparing saliency maps, which are also suitable for comparing flow direction maps: correlation-based measures, the Kullback-Leibler divergence, and the Receiver Operating Characteristic Analysis. These approaches can be used to, first, individually compute the similarities S_V and S_H between vertical and horizontal components of the flow direction maps; and, then, choose a norm for the vector 〈S〈=(S⃑_V,S_H ) that defines the similarity between the two scanpaths. In this paper, we provide an example of another more straight-forward approach that does not require the choice of a norm. We use cosine similarity cos θ in which θ is the angle between two direction vectors. Therefore, we can compute a similarity map. Each pixel’s value varies from −1 (opposite direction) to 1 (the same direction). We further apply a mask of the vectors’ magnitude (average of two flow direction maps) scaled to a range of (0, 1). This allows us to visualize only parts of the similarity map in which direction flows are important. Figure 9 shows a comparison of two participants’ scanpaths. We can notice that the upper part (blue areas) of the two scanpaths is rather different while the lower part (red areas) is similar. Notably, both scanpaths include a transition from the café terrace to the corner shop (cyan); and while participant A used a triangle pattern (as previously described), participant B mostly switched between the upper part and the center of the painting. More sophisticated approaches, such as a similarity measurement using global distributions [ 12 ], exist and can be used to compare the flow direction maps of different scanpaths.

## Conclusion and Future Work

In this paper, we illustrated the use of different visual aggregation techniques to obtain non-cluttered visual representations of scanpaths. Fixation clustering and saccade bundling simplified the scanpath representation and allowed the scan strategy of the participant to be read. Flow direction maps generated using edge bundling can be further represented as a single image to explore the transitions and can be compared using cosine similarity maps. Used together, these techniques provide an efficient support for a visual analysis of the scanpaths and informative illustrations of the eye movement data. We also provide the example datasets in the supplementary material so that other researchers can test their visualization methods on the data and compare it with our results.

It is worth noting that these are the first results based on observations of the rendered images. To further demonstrate the efficiency of such visualizations, it would be necessary to conduct a study with a group of participants to statistically validate our findings.

This work can be taken further in many directions. Using the proposed approach, we can visually simplify the scanpath of multiple participants. To do so, we will have to address the scalability issue with large quantities of data to be simplified. The used clustering and bundling algorithms have already proven capable of addressing these issues. The relative clutter of the generated layout despite their visual simplification can be further reduced using filtering based on the density map. For instance, we can choose to not display the least dense areas (which is partly done using the line width modulation), areas of some specific direction or time period. Finally, as addressed to some extent at the end of the discussion, quantitative metrics can be extracted from these simplified visualizations. Few metrics of scanpath comparison exist and our approach paves a new way to assess the eye tracking data.

### Ethics and Conflict of Interest

The author(s) declare(s) that the contents of the article are in agreement with the ethics described in http://biblio.unibe.ch/portale/elibrary/BOP/jemr/ethics.html and that there is no conflict of interest regarding the publication of this paper.
